# Navigating interstitial lung disease associated with rheumatoid arthritis (RA-ILD): from genetics to clinical landscape

**DOI:** 10.3389/fmed.2025.1542400

**Published:** 2025-02-24

**Authors:** Nicol Bernardinello, Margherita Zen, Gioele Castelli, Elisabetta Cocconcelli, Elisabetta Balestro, Raphaël Borie, Paolo Spagnolo

**Affiliations:** ^1^Respiratory Disease Unit, Department of Cardiac, Thoracic, Vascular Sciences and Public Health, University of Padova, Padova, Italy; ^2^Rheumatology Unit, Department of Medicine, University of Padua, Padova, Italy; ^3^Université Paris Cité, Inserm, PHERE, Hôpital Bichat, AP-HP, Service de Pneumologie A, Centre Constitutif du Centre de Référence des Maladies Pulmonaires Rares, FHU APOLLO, Paris, France

**Keywords:** interstitial lung disease, idiopathic pulmonary fibrosis, rheumatoid arthritis, genetics, *MUC5B*

## Abstract

Rheumatoid arthritis (RA) is a systemic autoimmune disease that affects millions of people worldwide and is characterized by persistent inflammation, pain, and joint destruction. In RA, the dysregulation of the immune system is well documented. However, the genetic basis of the disease is not fully understood, especially when extra-articular organs are involved. Interstitial lung disease (ILD) is a major cause of morbidity and mortality in patients with RA. Notably, RA-ILD shares several risk factors with idiopathic pulmonary fibrosis (IPF), namely male gender, smoking history, usual interstitial pneumonia (UIP) pattern of fibrosis, and association with the *MUC5B* rs35705950 polymorphism. In addition, other genetic susceptibilities are reported in RA-ILD for some HLA alleles and other less studied polymorphisms. However, the pathobiology of RA-ILD, particularly whether and to what extent genetic and environmental factors interact to determine the disease, remains elusive. In this review, we summarize and critically discuss the most recent literature on the genetics and pathogenesis of RA-ILD. The main clinical aspects of RA-ILD are also discussed.

## 1 Introduction

Rheumatoid arthritis (RA) is a systemic autoimmune disorder of unknown etiology that primarily causes erosive symmetric polyarthritis (1–3). RA represents a significant global healthcare burden, with an estimated prevalence of about 0.5–1% worldwide (1–3); the disease is more common in females than in males (4). Intriguingly, the prevalence and incidence of RA have risen during the last century, and currently, the disease affects more than 17·6 million people worldwide (5). Of note, the prevalence of the disease appears to decrease from North to South and from urban to rural areas (6, 7). The mechanism of RA is highly complex, involving inflammatory and autoimmune processes, and the primary trigger is unknown (8).

Broadly, RA is characterized by an insidious clinical onset with nonspecific symptoms, such as fatigue and sometimes fever, associated with pain, stiffness, and swelling in multiple joints. In most cases, the disease is progressive and gradually worsens, leading to uncontrolled inflammation that damages joint structures, including cartilage and bone (9, 10). The diagnosis can be made based on clinical features; in addition, serological biomarkers can be useful, including rheumatoid factor (RF) (11), which can test positive in 80% of people with RA, and antibodies to cyclic citrullinated peptides (ACPA) which can be found in 60 to 70% of patients (12).

Extra-articular manifestations (EAMs) of the disease occur in nearly 50% of all RA patients, and many organs, such as the spleen, lungs, and nervous system, can be affected (13). Lung involvement is one of the most frequent EAMs (60–80% of cases) (14). RA can virtually affect any part of the respiratory tract, from the pleura to blood vessels, with interstitial lung disease (ILD) representing the most frequent manifestation and a major determinant of prognosis (15, 16). However, who will develop ILD and what are the underlying mechanisms of this association remain largely unknown. Furthermore, it has been speculated that several genetics and environmental factors concur for developing ILD (17). With this background, this review aims to discuss the impact of ILD in patients with RA, highlighting the most recent evidence regarding genetic risk factors.

## 2 ILD in rheumatoid arthritis: an overview

**Figure 1:**
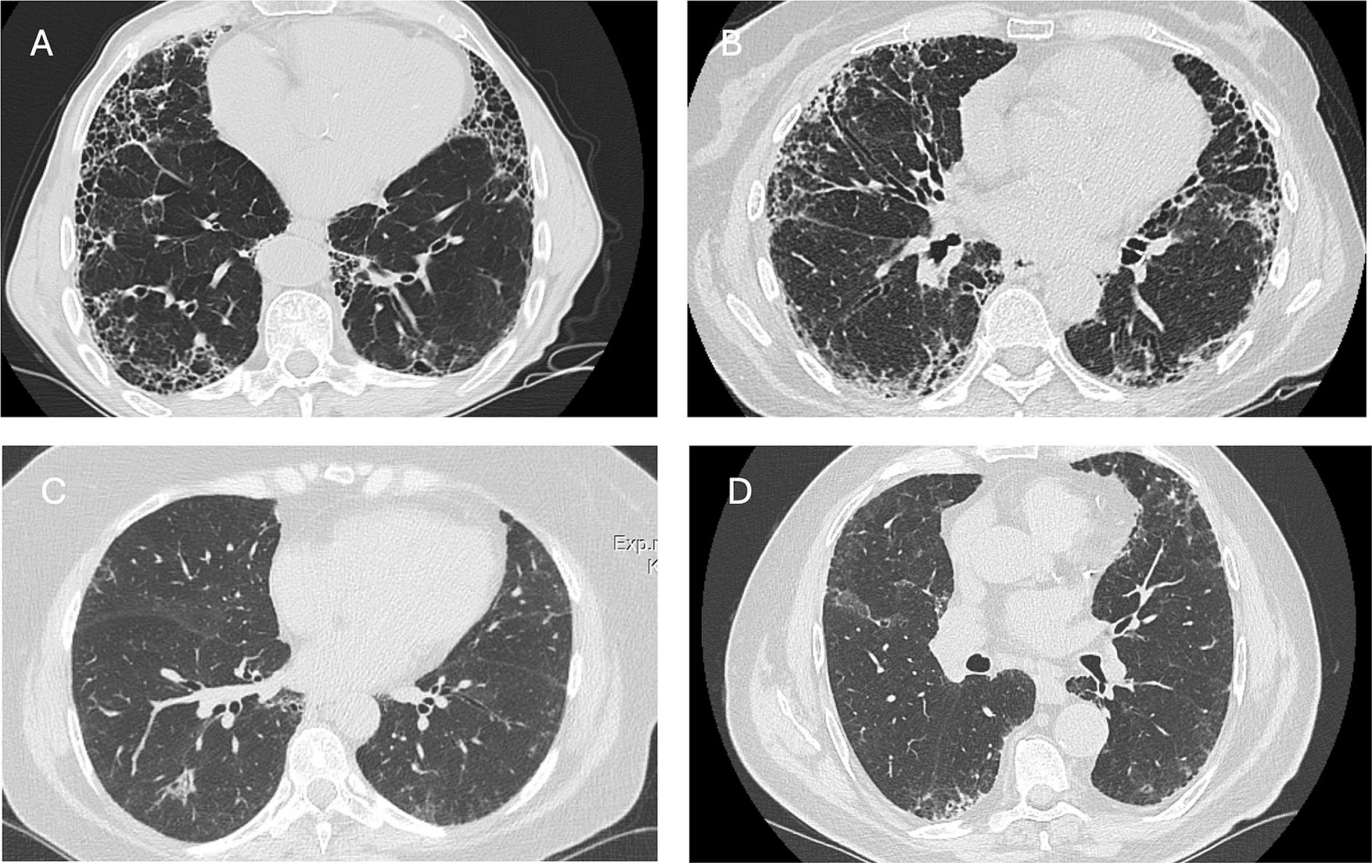
Axial CT scans illustrating the radiological presentation of patients with RA-ILD. **(A,B)** Usual interstitial pneumonia pattern in two male patients with rheumatoid arthritis. Honeycombing and traction bronchiectasis are visible in both patients. **(C)** Mild and migrant organizing pneumonia in female patients with rheumatoid arthritis. **(D)** Mild reticulation in an asymptomatic male patient with RA.

About 10% of patients with RA exhibit respiratory symptoms related to ILD (18–20). Notably, interstitial features on computed tomography (CT) scans have been found in up to 67% of cases, and lung involvement can sometimes occur before any articular manifestations (18, 21). Notwithstanding, the development of ILD is most prevalent in the initial years following the diagnosis of RA, although it can arise at any time during the course of the disease (22). Rheumatologists play a pivotal role in identifying ILD in RA: early suspicion is crucial, and the initial evaluation should involve a thorough chest examination and spirometry (23). It is essential also to remember that patients with RA without specific risk factors or respiratory symptoms can still be at risk of developing ILD (24). A high-resolution CT scan (HRCT) is necessary to detect lung involvement, characterize the pattern, and monitor disease evolution. More invasive diagnostic procedures, such as bronchoscopy or lung biopsy, are generally unnecessary and are only performed if diagnostic uncertainty persists. The usual interstitial pneumonia (UIP) pattern is the most frequently observed finding in RA-ILD and is characterized by reticulation, traction bronchiectasis, and honeycombing. The nonspecific interstitial pneumonia (NSIP) pattern is less common, while other patterns, such as organizing pneumonia (OP), are also seen but less frequently (25–27) (Figure 1).

Although RA-ILD can manifest with dry cough and dyspnea, some patients remain asymptomatic for a long time. Chest imaging is mandatory in all patients with abnormal findings on physical examination, such as “*velcro*”-like crackles or digital clubbing (28).

## 3 What we know about the genetic background in RA-ILD

In the last two decades, large-scale Genome-Wide Association Studies (GWAS) have revealed many common disease-associated variants in the context of different ILDs, especially in idiopathic pulmonary fibrosis (IPF), where the interest in genetic predisposition has steadily increased (29). Given the strong similarities with IPF, such as the presence of the UIP pattern, the same variants and polymorphisms have started to be investigated also in patients with RA-ILD (Table 1).

**Table 1:** Genetic variants and their impact on RA-ILD.

Gene	Impact on RA-ILD	References
MUC5B rs35705950 polymorphism	Well describe and connected with the risk of ILD in RA and UIP pattern in European population	Juge et al. (35) and Joo et al. (37)
Telomere length and telomere gene (TERT, TERC, RTEL1)	Associated with higher risk of ILD in RA Correlated with baseline disease severity	Natalini et al. (51), Doyle et al. (52), Tomos et al. (53), and Lee et al. (54)
HLA HLA-DRB1*16, DQB1*06 HLA-DRB1*04 and DQB1*04	HLA-DRB1*16, DQB1*06: risk factor for RA-ILD HLA-DRB1*04 and DQB1*04 protective for RA-ILD	Dedmon (58), Sharma et al. (59), Florescu et al. (62), and Furukawa et al. (63)
Other RPA3-UMAD1 rs12702634 PPFIBP2 FAM13A polymorphism rs2609255	Not reported in Europeans, reported in Japanese (controversial) Higher risk for RA-ILD Higher risk for RA-ILD	Shirai et al. (70), Juge et al. (71), and Higuchi et al. (72) Hayashi et al. (73) Jönsson et al. (64)

TERT, Telomerase reverse transcriptase; TERC, Telomerase RNA component; RTEL1, regulator of telomere elongation helicase 1; RPA3-UMAD1, UBAP1-MVB12-Associated (UMA) Domain Containing 1; PPFIBP2, PPFIA Binding Protein 2; FAM13A, Family with Sequence Similarity 13 Member A.

### 3.1 *MUC5B* rs35705950 polymorphism

*MUC5B* encodes a pivotal protein called Mucin-5B. It is one of the five gel-forming mucins in the saliva, cervical mucus, and lung (30). The *MUC5B* gain-of-function single nucleotide polymorphism in rs35705950 was deeply studied and is the strongest and the most known genetic risk factor for developing IPF (31, 32). Shortly, the rs35705950 polymorphism is associated with a 1.6 increased expression of Mucin-5B in the normal lung, which may impede a correct alveolar repair. Despite being associated with a higher risk of pulmonary fibrosis and some initial methodological discussion, recent studies demonstrated that in IPF patients, the rs35705950 T allele is associated with more prolonged survival (33, 34). In the last few years, the role of rs35705950 *MUC5B* has been investigated in patients with RA-ILD. Notably, Juge and coworkers identified that the *MUC5B* promoter variant was associated with an increased risk of ILD among patients with RA, particularly those with evidence of usual interstitial pneumonia pattern (UIP pattern) on high-resolution computer tomography (35). In the following years, the same authors advanced a new risk score that can help physicians identify RA patients at risk of subclinical ILD (36). The authors found a prevalence of subclinical RA-ILD of 19.0 and 16.9% in the two study cohorts. They also confirmed the role of the *MUC5B* rs35705950 T allele as independent risk factors for RA-ILD among the male sex, older age at RA onset, and increased disease activity in the joints (36). However, in a study conducted on 683 Korean patients, Joo and coworkers found no association between rs35705950 and risk of RA-ILD (odd ratio (OR) 2.49, 95% confidence interval (CI) 0.64 to 9.69, *p* = 0.187), probably because of the low prevalence of the mutant T allele in Asia. Nonetheless, they showed a significant association between the *MUC5B* rs35705950 T allele and UIP pattern (OR 4.90, 95% CI 1.23 to 19.59, *p* = 0.024) but not in patients with RA without ILD (37). All these studies shed light on the fact that there is a strict connection between this particular polymorphism and the specific radiologic pattern (i.e., UIP). Beyond this speculation, many authors also wondered if *MUC5B* polymorphism and the UIP pattern could represent a common denominator between RA-ILD and IPF. More in detail, the main question is: are we speaking about RA-UIP or IPF with RA? In a recent study conducted by Leavy et al., the authors demonstrated a considerable causal effect of IPF on seropositive RA, likely indicating that some mechanism associated with the development of UIP can promote RA (38). However, the connection between RA-ILD and IPF is currently under investigation, and future large multicenter studies are needed to better understand the intricate and complex connection between these two diseases (39, 40).

### 3.2 Telomere and telomere-related genes

Telomeres are described as the end part of chromosomes, and their role is to protect against DNA damage. In vertebrates, they consist of TTAGGG repeats (41). Consequently, the shortening of telomere length can lead to cell senescence and apoptosis in various ways, resulting in several immunological disorders and organ aging (42, 43). Telomere shortening has been deeply studied in IPF, and the presence of pathogenic variants in telomere-related genes, such as *TERT*, *TERC*, *PARN*, and *RTEL1* genes, are strongly associated with the risk of developing pulmonary fibrosis (44–46). However, the interest in telomere disorders in other diseases, such as COVID-19 (47, 48), has increased worldwide, with no exception on RA (49) and RA-ILD (50). In a landmark study, Juge et al. reported increased *TERT, RTEL1, PARN*, and *SFPTC* rare variants in patients with RA-ILD when compared with 1,010 control individuals of European ancestry, with an odds ratio (OR) of 3.17 and a 95% confidence interval (CI) of 1.53–6.12 (*p* < 0.001) (50). More recently, the association between telomere length and RA-ILD among a group of Veterans (54 RA-ILD patients and 92 RA-non-ILD patients) in the United States was investigated (51). The prevalence of ILD was significantly higher in patients with short vs. normal-length telomeres (73.3% vs. 32.8%, *p* = 0.002), and short telomeres were independently associated with increased odds of prevalent ILD compared to normal-length telomeres. Similar results were reported 1 year later in a study showing that shortened telomere length also correlated with baseline disease severity among RA-ILD patients (52). Telomere lengths were also compared between different ILDs and matched healthy controls (53). Seven patients with the RA-UIP ILD pattern appeared to have lower values compared with stable IPF, CPFE (combined pulmonary fibrosis and emphysema), and healthy controls [T/S (telomere index) mean 0.74 (±0.15) vs. 0.87 (±0.07) vs. 2.26 (±0.36)] (53). Very importantly, beyond the risk of ILD, telomere length has also been proposed as a potential new biomarker for the development of RA without ILD. It has also been suggested that short telomere length could enhance disease progression, and studies examining telomere length in RA have found that telomere erosion seems to occur more rapidly in subjects with RA than in healthy controls and that telomere lengths are shorter in those with the RA-risk Human Leukocyte Antigen (HLA)-shared epitope genes (54, 55). All these studies suggest that telomere shortening might contribute to the pathogenesis both for RA and RA-ILD (56).

### 3.3 Human leukocyte antigen

The Human Leukocyte Antigen (HLA) system is a complex of genes located in chromosome 6 (6p21.3). HLA is highly polymorphic, containing about 220 genes, many of which are pivotal for immune system coordination and the response against bacterial and viral infections (57). Class I contains the classic genes HLA-A, -B, and -C, while HLA class II comprises the HLA-DRB1, -DPB1, and -DQB1 genes. Some HLA-DR alleles are known to be linked with RA in different ethnic groups, while the DRB1 gene is known as the major genetic susceptibility locus for RA (58, 59). This was confirmed, above all, in patients who are positive for RF or ACPA (60, 61). In addition, some studies have stressed the association of extra-articular manifestations of RA with HLA-DR, including ILD. Of note, specific types of HLA (such as HLA-DRB1*16, DQB1*06, and DR2 serological group) were associated with a higher risk of ILD in RA. On the other hand, HLA-DRB1*04 (corrected P [Pc] = 0.0054, odds ratio [OR] 0.57), a shared epitope (SE) (*p* = 0.0055, OR 0.66), and DQB1*04 (Pc = 0.0036, OR 0.57) seems to be protective against interstitial lung involvement (62, 63).

### 3.4 Other emerging genes for RA-ILD

As previously mentioned, with the advent of next-generation sequencing (NGS), new opportunities were opened for genetics. In the specific context of RA-ILD, an interesting study was conducted by Jonsson et al. in 2022. In this project, 1,118 early RA patients were consecutively evaluated and followed prospectively; 60 (5.6%) had pulmonary fibrosis at the time of diagnosis or developed it during the disease course. 571,151 genome-wide single-nucleotide polymorphisms (SNPs) were ultimately analyzed. Four SNPs were associated with pulmonary fibrosis in RA: beyond the well-known *MUC5B* polymorphism rs35705950 and the *TERT* polymorphisms rs2736100, *TOLLIP* (rs111521887) and *FAM13A* (rs2609255) were also associated with a higher risk of ILD. In addition, older age and RF positivity were associated with an increased risk of ILD (64). The *TOLLIP* contributes to the toll-like receptors (TLR) pathways and is important for the endo-lysosome/autophagosome pathway in response to infections. The *TOLLIP* polymorphism has been deeply investigated in the pathogenesis and prognosis of idiopathic pulmonary fibrosis (IPF) and has been reported in small cohorts with conflicting results to be associated with treatment response (65–68). In the same way, the *FAM13A* variants have been associated with susceptibility to chronic lung diseases, also in IPF. In a study conducted by Hirano C. and coworkers, IPF patients who carry the T allele of the *FAM13A* polymorphism rs2609255 showed a significantly increasing mortality compared to the non-carriers (69).

The multitude of these studies suggests a strong connection between RA-ILD pathogenesis and IPF. Despite this speculation, some genetic associations may vary based on different ethnicities and must be evaluated carefully. For example, Shirai Y. et al. identified a novel RA-ILD risk locus at 7p21 that satisfied the genome-wide significance threshold (rs12702634 at *RPA3-UMAD1*, OR = 2.04, 95% CI 1.59 to 2.60, *p* = 1.5 × 10–8). The authors also found that the RA-ILD risk of the identified variant at *RPA3-UMAD1* was relatively high, specifically in patients with probable and definite UIP patterns (70). *RPA3* is a part of the heterotrimeric replication protein A complex (RPA), which plays an essential role in DNA replication and the cellular response to DNA damage, with a potential role in telomere maintenance. While this association seems supported in the Japanese population, the results found by Juge in 2024 were not in the same direction (71). In 883 European patients, the *MUC5B* rs35705950 was strongly associated with RA-ILD in all datasets, while no association between *RPA3-UMAD1* rs12702634 and RA-ILD was observed. Afterward, a Japanese study conducted by Higuchi and coworkers did not confirm the association between *RPA3-UMAD1* rs12702634 and RA-ILD, as previously documented by Higuchi et al. (72). The authors speculated that maybe both ethnicity and radiological pattern play a role in the development of the disease that seems extremely heterogeneous. However, further multicenter and international studies are needed to understand this complex connection between genes and ethnicity.

Another gene associated with RA-ILD is *PPFIBP2*. *PPFIBP2* is located on chromosome 11 in the intronic region of the gene encoding tyrosine phosphatase receptor type F polypeptide-interacting protein binding protein 2. *PPFIBP2* polymorphism rs6578890 seems to have the strongest association with ILD occurrence in 306 RA patients (73, 74).

In another study, the differential gene expression analysis revealed nine significantly upregulated genes in RA-ILD compared to RA without ILD: arginase 1 (*ARG1*), thymidylate synthetase (*TYMS*), sortilin 1 (*SORT1*), a marker of proliferation Ki-67 (*MKI67*), olfactomedin 4 (*OLFM4*), a baculoviral inhibitor of apoptosis repeat containing 5 (*BIRC5*), membrane-spanning 4-domains A4A (*MS4A4A*), C-type lectin domain family 12 member A (*CLEC12A*), and the long intergenic non-protein coding RNA (*LINC02967*). Interestingly, all these genes that are upregulated in RA-ILD vs. RA without ILD in this study are known to be involved in the pathogenesis of fibrosis. The authors also found that lower forced vital capacity (FVC) and DLCO and their decline over 6 months are associated with greater severity of RA-ILD (75). However, only a small number of patients were analyzed (*n* = 12), and the results presented can be managed only as a fundamental proof-of-concept.

Finally, also the polymorphisms rs11203366, rs11203367, rs874881 in *PADI4*, and rs1005753 in *PADI2* are associated with developing ILD in patients with RA. Furthermore, the SNPs rs1748033 in *PADI4*, rs2057094, and rs2076615 in *PADI2* were associated with RA development but not with ILD as well as haplotype in *PADI4* ACTC in patients with RA-ILD. Intriguingly, the levels of PAD4 protein are increased in ILD patients (76, 77).

## 4 Prognosis and survival of patients with RA-ILD

The clinical presentation of RA-ILD is highly variable, thus, new biomarkers and genetic variations that can predict the disease course are urgently needed. Of interest, some patients with RA-ILD develop progressive pulmonary fibrosis (PPF), characterized by an increasing worsening of the respiratory condition and consequently a higher risk of mortality (78–82). Although the development of ILD may have a substantial impact on RA prognosis, there is insufficient data for solid evidence-based guidelines. However, it is well known that RA-ILD is associated with a significant increase in mortality (83). In a US study of 582 patients, the risk of death over the follow-up period was almost 3-fold greater in patients with RA-ILD than in patients with RA alone (hazard ratio 2.86 [95% confidence interval (95% CI) 1.98–4.12]) (84). The same results were found in a prospective population-based study of 679 patients with RA-ILD and 11,722 matched patients with RA and no ILD. The 10-year mortality was 60.1% (95% CI 52.9–66.5) in the patients with RA-ILD, compared to 34.5% (95% CI 32.8–36.1) in the patients with RA and no ILD (22).

**Figure 2:**
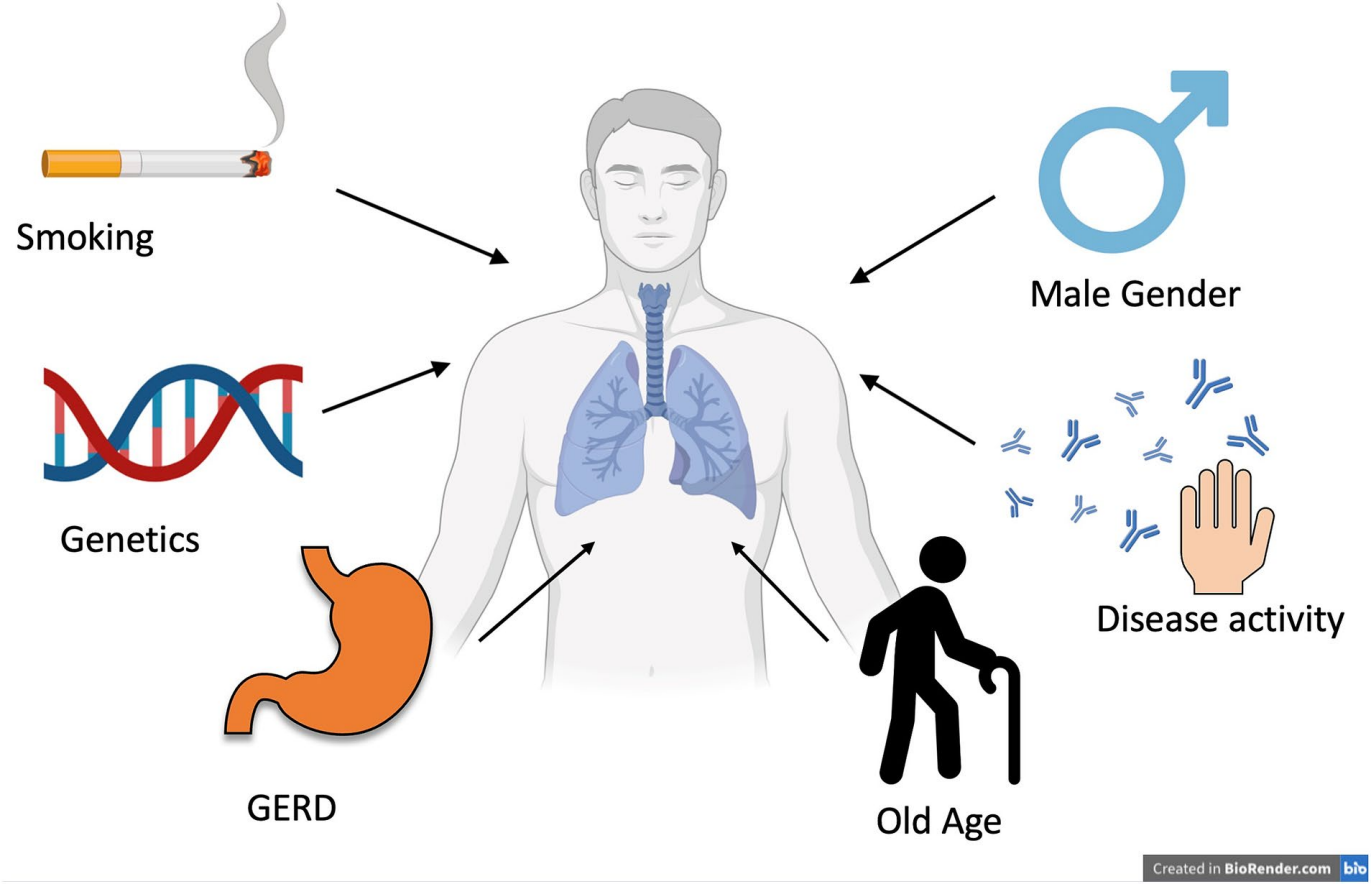
Risk factors associated with the development of ILD in patients with RA. GERD, gastroesophageal reflux. This picture summarizes the most frequent risk factors associated with the development of interstitial lung disease in patients with RA. This image was created by Biorender.com [Bernardinello (2025), https://BioRender.com/q67s977].

To facilitate early detection of RA-ILD, a Delphi-based consensus statement was recently proposed by a multidisciplinary team of pneumonologists, rheumatologists, and radiologists. The panel did not recommend screening all newly diagnosed RA patients. However, they strongly recommended the careful evaluation of patients with risk factors for RA-ILD, even in the absence of respiratory symptoms. Target risk factors are a history of smoking (especially more than 25 pack-years), male sex, older age, high disease activity (e.g., by a Clinical Disease Activity Index [CDAI] score > 10), and a family history of RA-ILD (85) (Figure 2). Additionally, the polymorphism of *MUC5B* was proposed as a new criterion. However, the predictive value of this risk score still needs validation in more extensive studies (86).

## 5 Conclusion

The occurrence of ILD in patients with RA is a complex and poorly understood process driven by different interacting factors, including genes and environment. However, many genes of varying effect size are likely to contribute to the predisposition to RA-ILD, although most of the reported associations await confirmation in larger studies. The identification of genetic determinants of ILD in RA is a pivotal step that would allow more precise risk stratification, while a deeper understanding of the disease pathobiology would facilitate the development of target therapies.
